# Randomized Controlled Trial of an Internet-Based Versus Face-to-Face Dyspnea Self-Management Program for Patients With Chronic Obstructive Pulmonary Disease: Pilot Study

**DOI:** 10.2196/jmir.990

**Published:** 2008-04-16

**Authors:** Huong Q Nguyen, DorAnne Donesky-Cuenco, Seth Wolpin, Lynn F Reinke, Joshua O Benditt, Steven M Paul, Virginia Carrieri-Kohlman

**Affiliations:** ^4^Office of Nursing ResearchUniversity of CaliforniaSan FranciscoCAUSA; ^3^Pulmonary and Critical CareUniversity of WashingtonSeattleWAUSA; ^2^Physiological NursingUniversity of CaliforniaSan FranciscoCAUSA; ^1^Biobehavioral Nursing and Health SystemsUniversity of WashingtonSeattleWAUSA

**Keywords:** Dyspnea, pulmonary disease, chronic disease, self-care, self-efficacy, health behavior, health education, exercise, monitoring, Internet, cellular phone, telemedicine, randomized controlled trial, Personal Digital Assistant (PDA)

## Abstract

**Background:**

People with chronic obstructive pulmonary disease (COPD) continue to experience dyspnea with activities of daily living (ADL) despite optimal medical management. Information and communication technologies may facilitate collaborative symptom management and could potentially increase the reach of such interventions to those who are unable to attend face-to-face pulmonary rehabilitation or self-management programs.

**Objective:**

The purpose of this randomized study was to test the efficacy of two 6-month dyspnea self-management programs, Internet-based (eDSMP) and face-to-face (fDSMP), on dyspnea with ADL in people living with COPD.

**Methods:**

We randomly assigned 50 participants with moderate to severe COPD who were current Internet users to either the eDSMP (n = 26) or fDSMP (n = 24) group. The content of the two programs was similar, focusing on education, skills training, and ongoing support for dyspnea self-management, including independent exercise. The only difference was the mode (Internet/personal digital assistant [PDA] or face-to-face) in which the education sessions, reinforcement contacts, and peer interactions took place. Participants returned to one of two academic clinical sites for evaluation at 3 and 6 months. The primary outcome of dyspnea with ADL was measured with the Chronic Respiratory Questionnaire. Secondary outcomes of exercise behavior, exercise performance, COPD exacerbations, and mediators, such as self-efficacy and social support, were also measured. A satisfaction survey was administered and a semistructured exit interview was conducted at the final visit.

**Results:**

The study was stopped early due to multiple technical challenges with the eDSMP, but follow-up was completed on all enrolled participants. Data were available for 39 participants who completed the study (female: 44%; age: 69.5 ± 8.5 years; percent predicted forced expiratory volume in 1 s: 49.6 ± 17.0%). The fDSMP and eDSMP showed similar clinically meaningful changes in dyspnea with ADL from baseline to 3 months (fDSMP: + 3.3 points; eDSMP: + 3.5 points) and sustained these improvements at 6 months (fDSMP: + 4.0 points; eDSMP: + 2.5 points; time effects *P* < .001; group by time *P* = .51). Self-reported endurance exercise time (*P* = .001), physical functioning (*P* = .04), and self-efficacy for managing dyspnea (*P* = .02) also showed positive improvements over time in both groups with no significant differences with respect to program modality. Participants who completed the study reported favorable satisfaction with the programs.

**Conclusions:**

Although there were numerous technical challenges with the eDSMP, both dyspnea self-management programs were effective in reducing dyspnea with ADL in the short term. Our findings will need to be confirmed in a larger randomized trial with more mature Web and personal digital assistant tools, use of a control group, and longer follow-up.

**Trial registration:**

clinicaltrials.gov NCT00102401, http://www.webcitation.org/5X8CX4gLC

## Introduction

Despite optimal medical therapy, people living with chronic obstructive pulmonary disease (COPD) continue to experience persistent dyspnea (shortness of breath) with their activities of daily living (ADL) and therefore must engage in the long-term tasks of self-management. Self-management has been defined as an “individual’s ability to manage the symptoms, treatment, physical and social consequences and lifestyle changes inherent in living with a chronic condition” [1]. Most interventions that support self-management are based on key principles of social cognitive theory [2] and are generally focused on increasing patients’ confidence in their ability to manage their illness and resulting symptoms by providing (1) relevant education so patients understand how their perception and behaviors can affect how much an illness interferes with their lives, (2) specific skills training and problem solving techniques, (3) goal setting and self-monitoring, and (4) sustained reinforcement of lifestyle changes [1,3-5].

Alternative care models, such as disease or care management programs, have been tested and shown to have some success in improving health outcomes in other diseases such as diabetes [6-8] and congestive heart failure [9,10], but programs for patients with COPD are yet to be implemented widely [11-13]. Pulmonary rehabilitation is a comprehensive evidence-based approach to supporting patients with COPD [14,15]. However, due to reimbursement policies in the United States, these programs are generally of short duration and may not be accessible to many patients because of distance, scheduling, and eligibility requirements. Thus, convenient and easy access to resources for self-management education, skills training, and ongoing support remains a notable challenge for patients with COPD and their caregivers. More recently, the pervasive increase in various forms of information and communication technology in everyday life provides a natural avenue and perhaps a partial solution for health providers to reach out to more patients and provide seamless support across the illness trajectory. Findings from a number of studies in the last several years have shown the positive impact of information and communication technology on health promotion and disease management activities in both healthy and clinical populations [16-22].

We previously tested a face-to-face dyspnea self-management program that combined individual education on strategies to decrease dyspnea with a home walking prescription, symptom monitoring, and telephone reinforcement by a nurse and found that this program decreased dyspnea with ADL over the long term [23]. The question remained whether an Internet-based program with similar components could bring about the same outcomes with greater reach to those who are not able to attend face-to-face programs. With the exception of a previous report on the feasibility and acceptability of engaging a small sample of patients with COPD in a nurse-facilitated, Web-based dyspnea self-management intervention by our group, there have been no other published studies on the use of the Internet for self-management support in this clinical population [24].

The purpose of this study was to extend our previous investigation by comparing the efficacy of the Internet-based dyspnea self-management program (eDSMP) with a face-to-face dyspnea self-management program (fDSMP) on the primary outcome of dyspnea with ADL in patients with moderate to severe COPD over a longer period using a randomized design. Secondary outcomes included exercise behavior, exercise performance, and COPD exacerbations. We hypothesized that the difference in changes in the primary outcome of dyspnea with ADL, measured by the Chronic Respiratory Questionnaire (CRQ), would not be greater than the minimal clinically important difference of 2.5 points between the two groups.

## Methods

### Study Design

We conducted a randomized, repeated measures (0, 3, and 6 months) pilot study to compare the effects of an eDSMP to an fDSMP in patients with COPD. The trial took place at two academic medical centers, University of California San Francisco, and University of Washington, Seattle. This research study was approved by the institutional review boards at both study sites and was registered with ClinicalTrials.gov (NCT00102401).

### Participants

Participants were recruited from a combination of Web-based and non-Web-based sources. Recruitment announcements were sent to various email distribution lists and online support groups for patients with COPD and older adults. Email postings were sent via a Web vendor intermediary who produced decision-support content for patients with COPD. Other recruitment activities included chest clinic referrals, letter mailings to university clinic patients with a COPD-related diagnosis, announcements at Better Breathers support groups and pulmonary rehabilitation programs, and newspaper advertisements.

The inclusion criteria were (1) a diagnosis of COPD and being clinically stable for at least 1 month; (2) spirometry results showing at least mild obstructive disease defined as post-bronchodilator forced expiratory volume in 1 s (FEV1) to forced vital capacity (FVC) ratio < 0.70 with FEV1 < 80% predicted, or FEV1/FVC < 0.60 with FEV1 > 80% predicted; (3) ADL limited by dyspnea; (4) use of the Internet and/or checking email at least once per week with a Windows operating system; (5) oxygen saturation > 85% on room air or ≤ 6 L/min of nasal oxygen at the end of a 6-minute walk test. Participants were excluded if they (1) had any active symptomatic illness (ie, cancer, heart failure, ischemic heart disease with known coronary artery or valvular heart disease, psychiatric illness, or neuromuscular disease); (2) participated in a pulmonary rehabilitation program in the last 12 months; or (3) were currently participating in > 2 days of supervised maintenance exercise.

### Randomization and Procedures

An investigator who was not involved in the day-to-day study operations generated the randomization sequence using the SPSS version 14.0 (SPSS Inc, Chicago, IL, USA) random sequence generator feature and placed the randomization in separate sealed opaque envelopes. The randomization scheme was stratified by the two clinical sites in blocks of six to ensure balanced allocation to the two treatment groups. Since registration and access to the Web questionnaires on the vendor-supported website required designation of a treatment group early in the baseline visit, the study nurse opened the randomization envelope during the first half of the visit. While the study nurse was privy to the treatment assignment, participants were not informed of their assignment until the visit was complete.

Baseline assessments included spirometry, completion of Web questionnaires, and 6-minute walk tests. Spirometry was performed using a Koko spirometer (Pulmonary Data Services, Louisville, CO, USA). Pulmonary function tests were used only to compare the severity of disease measured by airflow obstruction between the groups. At the end of the baseline visit, the study nurse introduced the personal digital assistant (PDA), a Blackberry 680, to the eDSMP participants; they were encouraged to play an electronic game on the PDA to increase their comfort with the device since it would be used to record their real-time symptom and exercise data. Participants assigned to fDSMP did not receive a PDA. All participants returned to the clinic within 1 week for an initial face-to-face dyspnea and exercise consultation with the study nurse coach and continued to participate in their respective intervention programs for the next 6 months. They returned to the medical center at 3 and 6 months for testing by study staff who were not involved in the intervention. Individual semistructured interviews were conducted either in person or via telephone at the final visit by the evaluation staff or investigators (HQN and VCK) who were not directly involved in the intervention.

### Dyspnea Self-Management Programs

#### Theoretical and Technical Framework

Major concepts from social cognitive [2,25], self-management [26], and pathophysiological [27] theories as well as our previous work on dyspnea self-management [23,28,29] provided the underlying framework for the dyspnea self-management program. Specifically, the dyspnea self-management program was comprised of education and skills training for dyspnea management, including individualized tailored exercise planning, self-monitoring of respiratory symptoms and exercise, and personalized reinforcement and feedback for exercising and the use of dyspnea self-management strategies. These programs were proposed to increase self-efficacy for exercise and dyspnea management. This improvement coupled with social support and possible physiological changes was hypothesized to ultimately reduce dyspnea with ADL and allow the dyspneic patient to increase his or her functional performance. Both programs were designed to provide similar content and “contact” time for ongoing reinforcement and support and differed only in the mode of delivery (Table 1). The eDSMP incorporated technological enhancements to support earlier recognition of worsening symptoms through real-time monitoring, more prompt feedback, and convenient access to information and support, which were hypothesized to attenuate the possible disadvantages of decreased face-to-face contact. 

We used a vendor-supported, Web-based application that was configured to our study specifications for the eDSMP [30] (see  Multimedia Appendix).

**Table 1:** Dyspnea self-management program components

Core Components	fDSMP	eDSMP
1. Dyspnea and exercise consultation (1-1.5 hours)	Individual face-to-face	Individual face-to-face Training on website and PDA
2. Endurance (4 times/week, 30 min/session) and arm strengthening (3 times/week) exercise program	Unsupervised independent exercise	Unsupervised independent exercise
3. Collaborative self-monitoring of exercise and respiratory symptoms and reinforcement of dyspnea management strategies (weekly in month 1; biweekly in months 2-6)	Paper diaries Reinforcement telephone calls (5-10 min)	PDA and Web diary Reinforcement emails
4. Structured education of dyspnea management strategies, skills training, and peer interactions (six 1-hour sessions)	Paper modules Face-to-face group sessions	Interactive Web modules Live group chat sessions Bulletin board

#### Dyspnea and Exercise Consultation

All participants returned to the clinic within 1 week of their baseline visit to participate in a 1.5- to 2-hour face-to-face consultation with an advanced practice nurse who specialized in either general adult or pulmonary medicine. The goal of the consultation was for the study nurse to establish rapport with the participant and to understand his or her current level of exercise and experience with dyspnea through motivational interviewing techniques [31]. An individualized exercise plan was developed with the participant, and actions that could be taken to prevent and manage future COPD exacerbations were discussed. The eDSMP participants were provided with a detailed paper help manual on how to navigate and use the website tools and their PDA. They received training on how to use the website to access the education modules, self-monitoring tools, and communication tools using the clinic computer. They also received training on how to record their daily exercise and symptoms using the PDA.

#### Exercise Program

During the consultation visit described above, the nurse and participant together developed an individualized exercise plan that was based on the participant’s baseline exercise performance, dyspnea at the end of a 6-minute walk test, oxygen saturation, stage of exercise motivational readiness, and exercise preferences. The home-based exercise program included a combination of endurance (walking, cycling, or swimming) and arm strengthening (biceps curls, triceps curls, side arm raises, and upper arm raises) exercises. All participants were encouraged to complete endurance exercises at least 4 times per week for 30 minutes per session and arm strengthening exercises at least 3 times per week. They used a modified 0- to 10-point Borg scale [32] to gauge their dyspnea as a proxy for exercise intensity and were instructed that they should feel at the end of their exercise that they could not have gone further. Participants who were more disabled were encouraged to perform their exercises in smaller 10-minute increments.

#### Collaborative Self-Monitoring and Reinforcement

**Figure 1:**
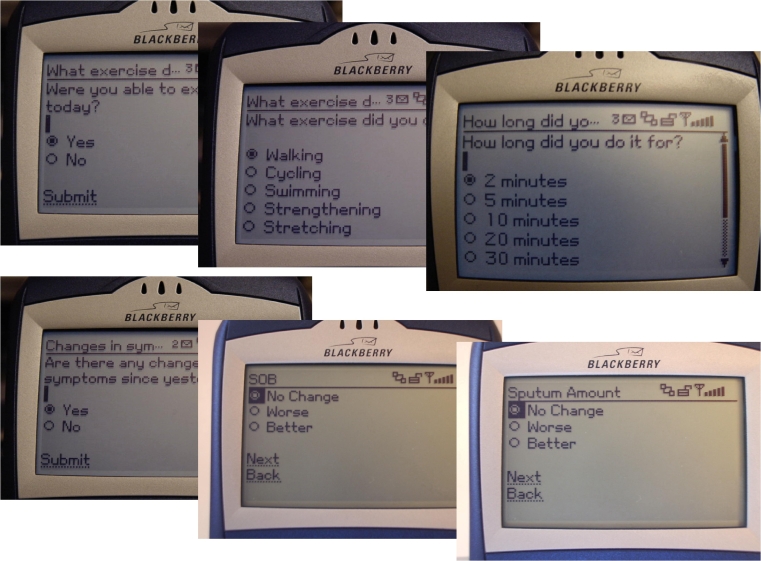
PDA exercise and symptom queries  (eDSMP group)

**Figure 2:**
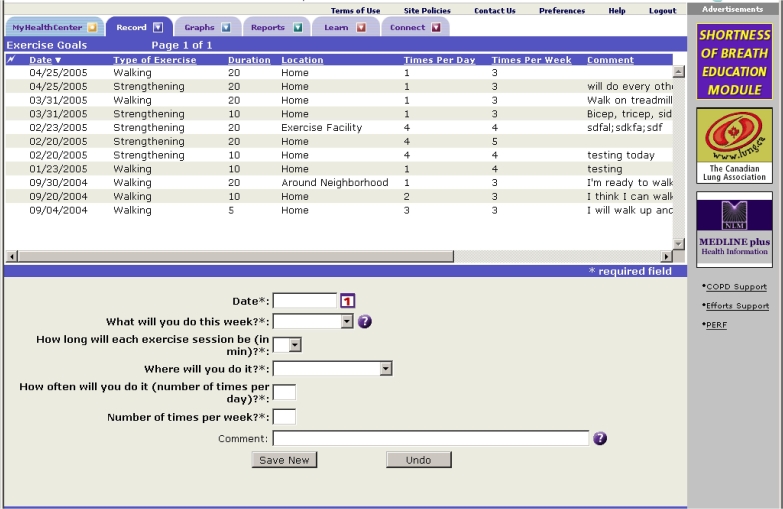
Exercise goal setting (eDSMP group)

The eDSMP participants submitted real-time information about their symptoms (dyspnea, sputum, sputum purulence, symptoms of a cold, wheezing, and cough) and exercise (mode, duration, and worst dyspnea) via the PDA (Figure 1) or website. The fDSMP participants completed paper diaries and mailed them back weekly to the study office. Participants in the eDSMP group were encouraged to communicate their exercise goals and progress to the nurse by using a Web-based goal-setting tool (Figure 2), whereas the fDSMP group set exercise goals during the telephone calls. The nurses reviewed this information to provide individualized feedback and reinforcement to participants regarding their use of dyspnea management strategies and exercise progress via email (eDSMP) or telephone (fDSMP), weekly for the first month and then biweekly for the next 5 months. These contacts were designed to be as similar as possible for the two groups. One difference was that automated email alerts were sent to the study nurses based on real-time symptom (worsening of symptoms from usual) and exercise (reports of not performing exercise for at least 3 consecutive days) data that the eDSMP participants submitted. There were no such alerts for the fDSMP participants.

#### Structured Education Sessions and Peer Interactions

All participants received education on shortness of breath (SOB), breathing strategies to reduce SOB, exercise and SOB, modifying activities to reduce SOB, coping with SOB and stress, and medications to manage SOB and COPD flare-ups. The eDSMP group accessed Web-based education modules, while the fDSMP participants were given a paper copy of the modules on these six topics. The Web modules, which were written at the 8th grade level or lower, also had nondigitized audio, pictures, and animations. The content from these modules was reinforced by study nurses during six weekly live chat sessions with participants from both clinical sites (eDSMP) or face-to-face meetings at the medical centers (fDSMP). These education sessions were designed to encourage peer interactions and mutual support.

### Outcome Measures

#### Primary Outcome

Dyspnea with ADL was measured with the CRQ-Dyspnea subscale, which has been validated in previous studies [33,34]. Participants chose five activities that were most important to them and were asked to rate the severity of dyspnea with these activities on a 7-point Likert scale ranging from “extremely short of breath” to “not at all,” with higher scores indicating less dyspnea. The benchmark for a minimal clinically important difference in mean scores is 2.5 [35]. Participants rated their dyspnea for these same activities at 3 and 6 months. We tested the concurrent validity of the Web-based CRQ-Dyspnea questionnaire by having 21 participants complete a paper version within one to seven days of the first administration during the baseline visit. While the individual responses for the five CRQ-Dyspnea questions were variable (*r* = 0.62), total scores were comparable (Web version: 15.7 ± 5.6 vs paper version: 15.1 ± 5.5).

#### Secondary Outcomes

##### Stage of Motivational Readiness for Exercise

Participants selected their readiness for exercise from a list of five descriptions (precontemplation, contemplation, preparation, action, and maintenance) [36].

##### Exercise Behavior

Participants were asked about the frequency and duration (5-, 10-, 20-, 30-, 40-, 60-minute increments) of endurance (walking, cycling, swimming), strengthening, and stretching (yoga, tai chi) exercises for a typical week during the last 4 weeks [24,37]. Total minutes per week with each type of exercise were calculated by multiplying the exercise frequency by session time in minutes.

##### Exercise Performance

Exercise performance was assessed using the 6-minute walk test. Subjects inhaled two puffs of a bronchodilator before the test. After standardized verbal instruction, two 6-minute walk tests were performed approximately 30 minutes apart on the same day in a hospital corridor [38]. Oxygen saturation, heart rate, and ratings of dyspnea were measured before and after both tests. The test with the greater distance was used in the analyses.

##### Health-Related Quality of Life

The CRQ and Medical Outcomes Study Short-Form 36 (SF-36) were used to measure disease-specific and general health-related quality of life (HRQOL), respectively. In addition to the dyspnea scale, the 20-item CRQ measures other components of disease-specific HRQOL, including fatigue, emotional functioning, and mastery (self-efficacy). The SF-36 has 36 questions that relate to nine distinct components of overall health and two composite measures of physical and mental functioning. Higher scores reflect better HRQOL for both instruments.

##### Acute COPD Exacerbations

 Acute COPD exacerbations were defined as an increase in any two major symptoms or an increase in one major and one minor symptom for at least two consecutive days and accompanied by a change in the medical regimen [39,40]. Major signs and symptoms included dyspnea, sputum volume, and sputum purulence; minor ones included symptoms of a cold (nasal discharge or congestion), wheezing, and cough. Participants provided daily ratings of these signs and symptoms either in the written logs (fDSMP) or their PDA (eDSMP) based on the following scale: no change, worse, or better [41,42].

#### Mediators of Treatment Effects

##### Knowledge

Knowledge of strategies to manage dyspnea was measured by a 15-item multiple choice and true/false questionnaire that was adapted from previously published instruments [43]. Internal reliability of the instrument was .72.

##### Self-Efficacy

Self-efficacy for managing dyspnea was measured using a single question with a 0- to 10-point response scale: “How confident are you that you can keep your shortness of breath from interfering with what you want to do?” [37].

##### Perception of Support

The information and emotional subscale of the Medical Outcomes Study Social Support Scale [44] was used to measure general perceived support. Questions related to exercise-specific support were modified from previous work [45] to assess participants’ perception of support from study nurses, family and friends, and health providers to either initiate or maintain an exercise program using a 7-point Likert scale ranging from “strongly agree” to “strongly disagree.”

##### Program Preference

Many participants volunteered their preference for either the fDSMP or eDSMP during the telephone screening. However, they were formally asked their preference during the dyspnea and exercise consultation by the study nurse.

##### Usage

Due to the configuration of the vendor’s Web server log files, we were unable to obtain detailed navigation information for each participant. We were, however, able to obtain proxy usage measures by virtue of timestamps recorded whenever eDSMP participants logged exercise and symptom data, set exercise goals, posted to the bulletin board, or participated in the chat sessions. Technical issues were documented and compiled.

##### Satisfaction

Participants were asked about their satisfaction with specific components of the eDSMP (13 items) or fDSMP (9 items) and their overall satisfaction with the programs using a 3-point scale (“not at all satisfied” to “very satisfied”). The evaluation staff or lead investigators (HQN and VCK) who were not directly involved with the interventions conducted semistructured interviews with participants at the end of the study. Participants were asked to provide feedback on what aspects of the program were most or least helpful for managing their dyspnea and how the program could have been done differently to support self-management. Probing questions were used to remind participants of the four major intervention components. Other questions were asked during this interview; however, a description of these questions is beyond the scope of the paper.

### Statistical Analyses

Independent *t* tests for continuous variables or chi-square and Fisher exact tests for categorical variables were used to compare baseline characteristics between the two groups. For all primary and secondary outcomes and mediators, we conducted repeated measures analysis of variance (ANOVA) tests that had one between-subjects factor— treatment group, with two levels (fDSMP and eDSMP)—and one within-subjects factor— time, with three levels (baseline, 3 months, and 6 months). This design allowed for testing the main effect of time, the main effect of treatment group, and the interaction of treatment group by time. We incorporated intent-to-treat principles whereby, for the participant who missed follow-up at 3 months (n = 1), baseline values were used, and for the participant who missed the follow-up at 6 months (n = 1), 3-month data were used. The intent-to treat analyses led to results that were comparable to those conducted using the available data only; therefore, results of the intent-to-treat analyses are reported. Since this was a pilot study with a relatively small sample size and all analyses of secondary outcomes were exploratory, we did not adjust the alpha levels for testing multiple outcome variables. Rather, we simply present the actual *P* values for each test. We did not examine differences in the outcomes between the two clinical sites since the samples were too small for meaningful comparisons. All statistical analyses were performed using SPSS version 14.0.

## Results

### Participants

**Figure 3:**
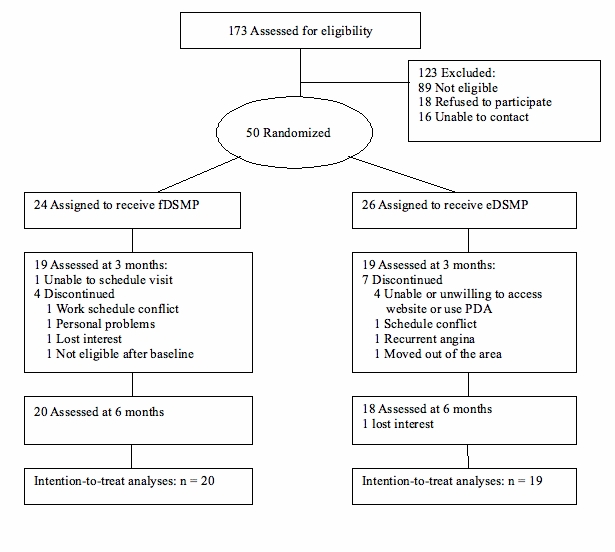
Subject flow

A total of 173 prospective participants were screened from April 2005 to July 2006 across both clinical sites. As shown in Figure 3, 50 participants were randomized to either the eDSMP (n = 26) or fDSMP (n = 24) arm after 123 participants were excluded (89 were not eligible, 18 refused to participate, and 16 were lost to contact). The investigators stopped the study early due to the cumulative technical and usability challenges that peaked when three consecutive eDSMP participants had multiple difficulties accessing the Web application and subsequently withdrew. All enrolled participants were followed through 6 months according to the study protocol.

Participants who dropped out after randomization (n = 11; 36% due to technical difficulties) were similar in age, education, employment status, distance from home to the respective clinical site, pulmonary functioning, disease severity (measured by the BODE composite index, which includes body mass index [BMI], FEV1, dyspnea, and 6-minute walk test) [46], stage of readiness for exercise, and treatment group preference compared to those who remained in the study. However, those who dropped out tended to be female (73% vs 44%, *P* = .09) and current smokers (27% vs 8%, *P* = .08), reported no musculoskeletal problems (0% vs 31%, *P* = .04), rated themselves as having advanced computer skills (55% vs 26%, *P* = .14), and were less likely to have participated in any face-to-face support groups (0% vs 28%, *P* = .05) or previously attended pulmonary rehabilitation (9% vs 44%, *P* = .04) compared with those who completed the study.

Participants in both treatment groups were similar on all baseline characteristics, suggesting that randomization was successful (Table 2). Approximately 66% of the sample expressed a preference for one of the two dyspnea self-management programs. There were notable differences between the groups in the proportion of participants who had a preference for either the fDSMP or eDSMP. Compared with only 25% of participants randomized to the fDSMP group who reported a preference for their assigned program, half of the participants randomized to the eDSMP group reported a preference for their assigned program (*P* = .01).

**Table 2:** Sample baseline characteristics*

	fDSMP (n = 20)	eDSMP (n = 19)	Total (n = 39)	Dropouts (n = 11)
**Demographics**				
Age, years (mean ± SD)	70.9 ± 8.6	68.0 ± 8.3	69.5 ± 8.5	67.3 ± 10.0
Female	9 (45%)	8 (39%)	17 (44%)	8 (73%)
Caucasian	20 (100%)	18 (95%)	38 (97%)	11 (100%)
Education				
High school or some college	8 (40%)	10 (50%)	18 (46%)	5 (45%)
College or more	12 (60%)	9 (50%)	21 (54%)	6 (55%)
Not currently employed or currently disabled or retired	15 (75%)	13 (72%)	28 (72%)	6 (55%)
Living situation: with spouse or other	13 (65 %)	12 (63%)	25 (64%)	6 (55%)
Currently smoking	1 (5%)	2 (11%)	3 (8%)	3 (27%)
Distance to clinical site, km (mean ± SD)	13.1 ± 15.7	20.4 ± 18	16.6 ± 17	10.4 ± 11.8
BMI, kg/m^2^ (mean ± SD)	27.7 ± 6.4	29.4 ± 5.9	28.5 ± 6.2	26.2 ± 4.2
				
**Disease Severity**				
FEV1/FVC (mean ± SD)	0.46 ± 0.11	0.49 ± 0.14	0.47 ± 0.13	0.48 ± 0.13
FEV1 % predicted (mean ± SD)	50.3 ± 17.6	49.0 ± 16.8	49.6 ± 17.0	52.8 ± 18.2
GOLD stage				
Mild/moderate	10 (50%)	9 (47%)	19 (49%)	5 (45%)
Severe/very severe	10 (50%)	10 (56%)	20 (51%)	6 (55%)
BODE composite score (mean ± SD)	2.8 ± 2.2	2.5 ± 1.5	2.7 ± 1.9	2.1 ± 1.6
Supplemental oxygen	5 (25%)	6 (33%)	11 (58%)	2 (18%)
Comorbidities				
Cardiovascular (HTN and CAD)	10 (50%)	9 (50%)	19 (49%)	4 (36%)
Musculoskeletal (arthritis and other pain)	8 (40%)	4 (22%)	12 (31%)	0 (0%)^†^
Previous pulmonary rehabilitation	8 (40%)	9 (47%)	17 (44%)	1 (9%)^†^
				
**Computer/Internet Skills**				
Self-rated computer skills				
Beginner	2 (10%)	4 (21%)	6 (15%)	2 (18%)
Intermediate	14 (70%)	9 (47%)	23 (59%)	3 (27%)
Advanced	4 (20%)	6 (32%)	10 (26%)	6 (55%)
Computer use, years (mean ± SD)	5.6 ± 2.7	5.7 ± 2.8	5.7 ± 2.7	6.4 ± 2.9
Hours on the Internet per week, median (range)	9.5 (1-25)	15.0 (1-90)	14 (1-90)	12 (2-35)
Participate in online support groups	2 (10%)	5 (28%)	7 (37%)	1 (9%)
				
**Other Characteristics**				
Motivational readiness for exercise				
Precontemplation/contemplation	6 (30%)	8 (42%)	14 (36%)	3 (27%)
Preparation	8 (40%)	6 (33%)	14 (36%)	5 (46%)
Action/maintenance	6 (30%)	5 (28%)	11 (28%)	3 (27%)
Treatment group preference^‡^				
fDSMP	5 (25%)	7 (39%)	12 (31%)	4 (50%)
eDSMP	4 (20%)	10 (50%)	14 (36%	3 (38%)
No preference	11 (55%)	2 (11%)	13 (33%)	1 (12%)
Outcome expectation of dyspnea self-management program^§^				
Very/extremely	11 (55%)	11 (58%)	22 (56%)	5 (46%)
Quite	3 (15%)	6 (32%)	9 (23%)	4 (36%)
Fair	6 (30%)	2 (10%)	8 (21%)	2 (18%)

^*^Values are number (%) unless otherwise stated. BMI, body mass index; FEV1/FVC, forced expiratory volume in 1 s/forced vital capacity; GOLD, Global Initiative for Obstructive Lung Disease; BODE, body mass index, airflow obstruction, dyspnea, exercise; HTN, hypertension; CAD, coronary artery disease.

^†^ *P* < .05 (dropouts vs participants).

^‡^ *P* = .01 (fDSMP vs eDSMP).

^§^“How much do you think this program (eDSMP or fDSMP) will assist you in managing your shortness of breath?” (1- to 6-point Likert scale: “not at all” to “extremely”).

### Outcomes

#### Primary Outcome: Dyspnea with ADL

While there was a significant main effect of time (*P* < .001), the lack of a significant group by time interaction (*P* = .51) indicates that the trajectory of change in dyspnea with ADL over time was not different between the two programs (Table 3). Participants in both programs showed similar clinically meaningful changes in dyspnea with ADL from baseline to 3 months (fDSMP: + 3.3 points; eDSMP: + 3.5 points) and, for the most part, sustained these improvements at 6 months (fDSMP: + 4.0 points; eDSMP: + 2.5 points).

**Table 3:** Comparison of treatment effects: dyspnea, exercise, exercise performance, and HRQOL*

	fDSMP (n = 20)	eDSMP (n = 19)	Group *P* Value	Time *P* Value	Group × Time *P* Value
**Primary Outcome**					
CRQ-Dyspnea with ADL (score range: 5-35)^†^					
Baseline	15.9 ± 5.4	18.8 ± 6.2	.14	< .001	.51
3 Months	19.2 ± 5.8	22.3 ± 4.6			
6 Months	19.9 ± 6.2	21.3 ± 6.0			
					
					
**Secondary Outcomes**					
Exercise stage of change: action or maintenance					
Baseline, no. (%)	6 (30%)	5 (26%)	-	-	-
3 Months, no. (%)	14 (70%)	16 (84%)			.47^‡^
6 Months, no. (%)	15 (75%)	12 (63%)			.85^‡^
Endurance exercise (total min/week)					
Baseline	77 ± 113	89 ± 102	.22	.001	.99
3 Months	141 ± 100	173 ± 130			
6 Months	121 ± 81	128 ± 111			
Strengthening exercise (total min/week)					
Baseline	21 ± 46	11 ± 29	.54	< .001	.61
3 Months	56 ± 66	53 ± 70			
6 Months	53 ± 59	34 ± 37			
6-Minute walk test (m)^§^					
Baseline	406 ± 150	436 ± 92	.22	.70	.05
3 Months	386 ± 157	450 ± 91			
6 Months	394 ± 165	456 ± 91			
					
CRQ-Fatigue (score range: 4-28)^†^					
Baseline	16.1 ± 4.4	17.1 ± 5.3	.29	.03	.13
3 Months	16.6 ± 4.8	19.4 ± 4.1			
6 Months	17.7 ± 5.2	18.3 ± 4.4			
CRQ-Mastery (score range: 4-28)^†^					
Baseline	20.4 ± 5.2	21.7 ± 3.2	.35	< .001	.98
3 Months	22.3 ± 5.8	23.6 ± 2.9			
6 Months	22.4 ± 5.5	23.6 ± 3.7			
CRQ-Emotional functioning (score range: 7-49)^†^					
Baseline	33.4 ± 8.0	35.9 ± 7.2	.33	.38	.98
3 Months	34.6 ± 8.7	36.8 ± 7.5			
6 Months	34.5 ± 8.6	36.8 ± 7.8			
CRQ-Total score (score range: 20-140)^†^					
Baseline	85.8 ± 18.9	93.5 ± 15.7	.19	< .001	.60
3 Months	92.7 ± 22.5	102.1 ± 15.6			
6 Months	94.5 ± 22.6	99.9 ± 16.8			
					
SF-36 Physical composite (score range: 0-100)^†^					
Baseline	32.8 ± 8.5	37.3 ± 7.0	.07	.04	.99
3 Months	35.3 ± 11.0	41.0 ± 7.9			
6 Months	35.2 ± 10.6	39.9 ± 7.6			
SF-36 Mental composite (score range: 0-100)^†^					
Baseline	51.8 ± 9.9	49.7 ± 10.1	.70	.31	.47
3 Months	52.2 ± 11.7	52.8 ± 9.6			
6 Months	53.5 ± 11.6	51.3 ± 10.0			

^*^Values are mean ± SD unless otherwise stated. CRQ, Chronic Respiratory Questionnaire; ADL, activities of daily living; SF-36, Medical Outcomes Study Short-Form 36.

^†^Higher scores are better.

^‡^Chi-square test.

^§^For the eDSMP group, n = 18.

#### Secondary Outcomes: Exercise Behavior, Exercise Performance, HRQOL, and Acute Exacerbations of COPD

A majority of participants in both groups advanced in their stage of readiness for exercise with up to 84% reporting that they were in either action or maintenance at 3 months (see Table 3). This was consistent with changes in total duration of endurance exercise per week from baseline to 3 months, + 84 mins (eDSMP) and + 64 mins (fDSMP), and at 6 months, + 39 mins (eDSMP) and + 44 mins (fDSMP). However, exercise performance as measured by distance covered during the 6-minute walk test declined in the fDSMP and increased in the eDSMP over time with a marginal group by time difference (*P* = .05).

Total scores on the CRQ, reflecting disease-specific HRQOL, improved over time for participants in both the eDSMP and fDSMP (*P* < .001). There were also positive changes in the SF-36 physical composite scores over time for both groups (*P* = .04). Neither of the programs had an impact on the SF-36 mental health composite score.

There was a total of 11 acute exacerbations of COPD in 10 participants, captured either through the electronic or paper diaries or obtained during the telephone or email follow-up contacts. The short study duration and heterogeneous disease severity across participants made it unrealistic to capture enough events for group comparisons.

#### Mediators of Treatment Effects: Knowledge, Self-Efficacy, Perception of Support, Program Preference, Usage, Technical Issues, and Satisfaction

There were small improvements in participants’ already high baseline knowledge of dyspnea management strategies at 3 months, which was sustained at 6 months (*P* < .001), with no group differences over time (*P* = .68; Table 4). Participants in both programs improved their self-efficacy for managing dyspnea (*P* = .02) with no group by time differences. These positive changes were also captured in the CRQ mastery subscale (*P* < .001; see Table 3). Perception of general social support did not appreciably change (*P* = .42) or differ between groups over time (*P* = .68). However, participants reported that they agreed or strongly agreed that they received the support from the study nurses needed to either start or maintain their exercise programs (3 months: fDSMP, 91%; eDSMP, 100%; 6 months: fDSMP, 90%; eDSMP, 100%).

**Table 4:** Comparison of mediators of treatment effects: knowledge, self-efficacy, and perception of support*

	fDSMP (n = 20)	eDSMP (n = 19)	Group *P* Value	Time *P* Value	Group × Time *P* Value
**Knowledge**					
Dyspnea knowledge (score range: 0-15)^†^					
Baseline	12.5 ± 2.3	12.6 ± 1.8	.49	< .001	.68
3 Months	13.3 ± 1.6	13.8 ± 1.0			
6 Months	13.8 ± 1.5	14.1 ± 1.0			
**Self-Efficacy**					
Self-efficacy for managing dyspnea (score range: 0-10)^†^					
Baseline	4.6 ± 2.4	4.7 ± 2.3	.18	.02	.34
3 Months	5.5 ± 3.3	6.8 ± 2.3			
6 Months	5.0 ± 3.6	6.7 ± 2.6			
**Perception of Support**					
Perception of general social support (score range: 0-100)^†^					
Baseline	68.9 ± 37.2	62.2 ± 27.6	.64	.42	.68
3 Months	65.2 ± 31.7	64.0 ± 24.3			
6 Months	70.9 ± 31.0	66.4 ± 27.1			
Perception of exercise support from research staff ^‡^					
3 Months			-	-	-
Strongly agree, no. (%)	13 (65%)	14 (74%)			
Agree, no. (%)	5 (26%)	5 (26%)			
6 Months			-	-	-
Strongly agree, no. (%)	16 (80%)	13 (68%)			
Agree, no. (%)	2 (10%)	6 (32%)			

^*^Values are mean ± SD unless otherwise stated.

^†^Higher scores are better.

^‡^At 3 months, n = 19 for fDSMP group.

Approximately 38% (n = 15) of the participants were randomly assigned to their preferred program; 28% (n = 11) were assigned to their nonpreferred program. The remaining 13 participants expressed no program preference. Comparisons across these three groups (concordant, discordant, nonpreferential) on the binary outcome of change in the CRQ-Dyspnea of at least + 2.5 points showed no differences among the groups in the proportion of participants who met this clinically important improvement threshold at 3 or 6 months (*P* = .40 and .39, respectively). Participants who preferred the eDSMP tended to be younger (65 ± 8 vs 72 ± 7 vs 72 ± 9 years, *P* = .06), lived further away from the clinical site (24 ± 21 vs 15 ± 16 vs 11 ± 9 km, *P* = .12), and rated their computer skills as advanced (43% vs 17% vs 15%, *P* = .05) compared to those who preferred the fDSMP or had no program preference, respectively; there were no other notable differences across the preference groups.

A majority of the technical issues for the eDSMP had to do with access to the study website. Participants had to install proprietary security software plug-ins in order to access the site. Five participants had at least two pop-up blocker software programs on their systems and required remote assistance from the vendor’s technical support staff to disable the programs. Three participants expressed concerns about disabling their pop-up blocker software and security vulnerabilities when accessing the site with the Internet Explorer browser; the site was not accessible with non-Windows-based operating systems or other Web browsers. One participant required almost 5 hours of technical support from the vendor before she could access the site. Participants commented during the exit interview that the decreased accessibility, slow loading of the Web application, and security concerns discouraged them from using the site more regularly.

There were also notable usability challenges with the wireless-enabled PDA and unreliable wireless coverage [47]. Participants had to complete 16-30 unique actions on the device to submit an exercise or symptom entry. When asked about the least helpful component of the study, one participant commented, “The most annoying was the blackberry [PDA]. If you exercised three different ways, for example, cardio, weights, and stretching, you had to go back through the symptoms questionnaire for each type of exercise.” Another commented that he changed his reporting behavior once he learned of the branching logic for the symptom surveys: “I would answer ‘no change’; it was too bothersome to report change since I would then have to go through each of the screens.” Inconsistent wireless coverage was also problematic: “The PDA did not allow me to document [my data] when I left the city. The technical glitches need to be fixed. It worked well when it worked.”

The numerous technical problems decreased participant engagement with the Web and PDA tools, and this was reflected in the number of Web log-ins and the exercise and symptom entries via the website and/or the PDA (Table 5). The exercise goal-setting tool and bulletin board were seldom used by eDSMP participants. One participant who was initially interested in using the bulletin board for peer-to-peer communication expressed his disappointment: “The bulletin board—no one uses it to ask questions.” Only two eDSMP participants used the exercise goal-setting tool more than five times. When probed about use of specific tools on the site, one participant commented, “I never remembered to do the goal setting or graphing on the website.”

A total of 77 and 122 exception alerts were generated based on lapses in exercise entries or reports of worsening symptoms from usual, respectively. Most fDSMP participants (80%) attended all six face-to-face education sessions (5.8 ± 0.6 sessions), while more of the eDSMP group (96%) participated in at least six online chat education sessions (6.2 ± 2.0 sessions). The number of email and telephone reinforcement follow-ups was comparable between groups.

Participants in both groups were most satisfied with the initial face-to-face interviews (Table 6). Use of the PDA and peer interaction received the lowest ratings by the eDSMP group. Mean ratings of overall satisfaction were only slightly lower in the eDSMP compared to the fDSMP group.

**Table 5:** Usage statistics over 6 months for eDSMP

Usage Parameter	Mean ± SD (Range)
Website log-ins	59 ± 34 (20-151)
Exercise goal setting	4 ± 6 (0-25)
Exercise entries	156 ± 80 (51-338)
Symptom entries	137 ± 48 (17-229)
Exercise exception alerts	4 ± 5 (0-17)
Symptom exception alerts	6 ± 6 (1-20)
Reinforcement emails	14
Education sessions	6.2 ± 2.0 (0-11)

**Table 6:** Satisfaction with the dyspnea self-management program*

	fDSMP (n = 20)	eDSMP (n = 19)
Initial face-to-face interview	2.9 ± 0.31	2.8 ± 0.48
Education sessions	2.7 ± 0.71	2.4 ± 0.78
Educational materials	2.6 ± 0.68	2.6 ± 0.50
Exercise goal setting	2.6 ± 0.59	2.4 ± 0.70
Exercise and symptom self-monitoring	2.5 ± 0.69 (paper log)	2.1 ± 0.73 (PDA) 2.4 ± 0.69 (website)
Receiving exercise prompts on PDA	N/A	2.4 ± .51
Reinforcement (telephone vs email)	2.7 ± 0.66 (telephone)	2.6 ± 0.50 (email)
Interaction with peers	2.2 ± 0.86	1.9 ± 0.80
Assistance with managing acute exacerbations of COPD	2.3 ± 0.73	2.4 ± 0.78
Overall program	2.7 ± 0.47	2.6 ± 0.51

^*^1 = not at all satisfied, 2 = quite satisfied, 3 = very satisfied. Values are mean ± SD.

## Discussion

We found that older adults with moderate to severe COPD showed clinically and statistically meaningful improvements in dyspnea with ADL as a result of participating in either a 6-month, face-to-face (fDSMP) or an Internet-based (eDSMP) dyspnea self-management program. These changes were consistent with overall increases in the mediator of self-efficacy for managing dyspnea and in the secondary outcomes of self-reported exercise endurance time and physical functioning. This is the first study we are aware of that employed a randomized design to test the effects of a technology-enhanced dyspnea self-management intervention for patients with COPD.

This study builds on our previous published findings with the fDSMP [12,23] and, more recently, the eDSMP [24]. Our overall goal is to be able to offer two comparably effective programs to broaden the reach to help more patients with COPD manage their dyspnea. As such, both programs were designed to provide similar content and contact time and only differed with regard to the mode that was used for education, collaborative self-monitoring, reinforcement, and peer interaction. It is noteworthy that participants in the eDSMP experienced reductions in their dyspnea despite considerable technical and usability challenges with our Web-based desktop and PDA application. The eDSMP participants who completed the study generally reported a positive experience with the program, especially their interactions with the study nurses, despite the technical challenges with accessing the website and using the PDA. These findings suggest that the “active ingredient” of the eDSMP probably had less to do with the technology and more to do with the ongoing feedback and focused motivational support on dyspnea self-management they received from the nurses via email and during the online educational chat sessions. We hypothesize that the initial face-to-face dyspnea and exercise consultation was also probably critical in fostering a positive relationship between eDSMP participants and study nurses.

For participants who were able to log their exercise and symptoms using their desktop computer or PDA, the study nurses could review this information in real time and provide feedback and encouragement. Even for those who had trouble with either the website or PDA, the nurses showed a genuine interest in the participants’ well-being and consistently used motivational techniques to reinforce their confidence in self-management of dyspnea, including regular exercise. We believe that these positive nurse–patient collaborative interactions that were not dependent on the Web application and primarily occurred asynchronously via email increased the eDSMP group’s engagement in exercise and consequently provided a positive impact on the perception of dyspnea similar to that of the fDSMP. Our observations are in line with findings from other behavioral studies of Internet-based physical activity and weight loss interventions. Those programs in which participants corresponded with and received regular feedback from a human counselor had increased treatment adherence that resulted in more robust outcomes [20,21,48,49]. Tate et al [21] tested the efficacy of a self-directed Internet weight loss program compared with a similar program that was supplemented with behavioral counseling either from an automated expert system or a human counselor. While weight loss was comparable between the two active arms at 3 months, the group that received feedback from a live counselor had significantly greater weight loss at six months. The study by Wing and colleagues [20] suggests that an Internet-based weight maintenance program, which included use of human counselors, was as effective as a face-to-face program in decreasing the number of participants who regained weight. Earlier studies of Internet-based behavioral interventions that did not include face-to-face contact or interactions from a human interventionist had weak effects [50,51]. Interestingly, a recent study of healthy middle age and older adults showed that those who received automated physical activity counseling advice via telephone had similar improvements in self-reported physical activity over 12 months compared to the group that received human advice [52]; both groups received an initial in-person exercise counseling session with a trained health educator.

A number of the participants in the current study reported that they enrolled in the study because they desired to “stay accountable to something or someone” and that they would be less likely to exercise if they were not “monitored.” This theme was also reported in a recent study of an Internet-based physical activity program with healthy adults [53]. These observations may reflect the attitudinal characteristics specific to participants who seek out and volunteer for these types of research studies. An important question to address in future studies is whether this sense of accountability and commitment could be maintained with less resource-intensive approaches. Although there are no published cost-effectiveness analyses of Internet-based behavior change interventions, it would seem that interventions like our dyspnea self-management program, which include an empathetic and caring health provider, could perhaps reach more patients; however, they may be no more cost-effective than face-to-face programs. Economic evaluations of different models of Internet-based interventions for chronically ill older adults will need to be conducted before such resource-intensive interventions can be scaled up to the population level.

Patient-centered models of care suggest that health care should be “tailored” to the individual and provided in accordance with their values and preferences [54]. Thus, it is particularly important that investigators testing different delivery channels assess participant preferences and examine whether these preferences actually moderate participation and response. The study nurses observed that some participants preferred certain aspects of both programs (ie, telephone calls rather than email, but chat room rather than in-person education sessions). We measured participant preferences and found that concordance between program preference (eDSMP or fDSMP) and program assignment did not result in greater improvements in the primary outcome of dyspnea. A weakness of our study and others that test for interactions between delivery channel preferences and improvements in outcomes is the small sample sizes. Future studies will need to be adequately powered to examine how individual preferences, perhaps measured at different times during the study, modify participant engagement in the intervention and affect outcomes. In addition, a greater understanding of the factors that shape participants’ preferences for different modes of communication may help to identify mechanisms that increase acceptability, participant engagement, and retention.

### Limitations

Several limitations must be considered in interpreting our study findings. While the results are encouraging, it is important to note that due to significant technical and usability challenges, which for the most part could have only been resolved with a complete redesign of the Web and PDA application, it was necessary to stop the study early. We nevertheless gleaned important insights from this pilot study on the role of information and communication technologies in supporting collaborative self-management with older chronically ill patients and methodological issues that would have to be addressed with such clinical studies in the future [55].

Since the primary study outcome is a symptom and can only be derived through self-report, we have to assume that what we captured was the best representation of participants’ dyspnea experiences. While changes in dyspnea with ADL for both groups were accompanied by changes in other conceptually similar self-reported measures (eg, self-efficacy for dyspnea management and physical functioning), we did not observe improvements in a more objective indicator—distance covered during a 6-minute walk test. These observations are similar to that of our earlier study of the fDSMP in which dyspnea with ADL decreased but with only small changes in exercise performance [23]. However, the findings are in contrast to pulmonary rehabilitation programs in which exercise performance usually improves in conjunction with reductions in dyspnea with ADL [15]. Based on our theoretical framework, the dyspnea self-management programs could be acting through a different pathway to reduce dyspnea with ADL (eg, increased confidence, cognitive reframing, or activity modification instead of increased fitness, which is typically associated with higher intensity supervised exercise training interventions).

It is possible that study participants desired to impress the investigators by responding favorably to the self-reported measures. We doubt that this was the case. Approximately one third of the sample was already in the active or maintenance stage of exercise and reported engaging in an average of 83 minutes of exercise per week at baseline, which is surprisingly comparable to a recent report on a large sample of patients with COPD [56]. With such relatively high levels of physical activity, one might expect a regression to the mean, but instead, endurance and strengthening exercise increased in both groups to levels that met or exceeded public health guidelines for physical activity [57]. Moreover, we worked with participants to incorporate upper extremity strengthening exercises that theoretically would improve dyspnea with ADL. These exercises may not necessarily have an impact on walking performance. Many ADL involve arm activities, and in COPD, upper extremity activities produce substantial dyspnea. Regardless, it will be important for future studies to include objective assessments of free living physical activity since this is one parameter that can easily be triangulated with self-report [58].

Due to the technical and usability challenges with the Web and PDA application and differential participant attrition, we terminated the study before reaching our sample target. The absence of a significant group by time effect in the changes in the primary outcome of dyspnea could be due to insufficient power. Nevertheless, the differences in the dyspnea change scores between the two programs were small and not of the magnitude that would meet the accepted benchmark for a clinically significant difference. Future studies will need to confirm whether these two programs can indeed produce and sustain such benefits beyond 6 months and are superior to a control intervention.

Study participants were primarily Caucasian and generally well educated, reflecting the demographics of early Internet adopters [59]. These characteristics make the findings less generalizable to the broader population of COPD patients. Although we excluded participants who had completed pulmonary rehabilitation within the last 12 months, 42% of the participants had previously participated in pulmonary rehabilitation. This reflects a sample that is generally more engaged and motivated since only a small percentage of patients with COPD ever participate in pulmonary rehabilitation [56].

### Conclusion

Despite these limitations, this is the first study we are aware of that employed a randomized design to test the effects of a technology-enhanced dyspnea self-management intervention for older patients with COPD. The study included objective verification of disease severity with spirometry and exercise performance testing, which are often absent from eHealth studies. The sustained improvements in dyspnea with ADL over repeated measurements reflect the specificity of the intervention, that of dyspnea management. If future studies confirm that the two programs can effect significant improvements in dyspnea with ADL and secondary health outcomes, the potential for use in the continuum of self-management interventions is enormous (eg, symptom management for patients with other cardiopulmonary diseases or those with mild disease who are not eligible for pulmonary rehabilitation, a “booster” for graduates of these programs, or as palliative care for those who are too ill to participate in face-to-face programs).

## Multimedia Appendix

Selected PDA and website screenshots

## Abbreviations

ADL: activities of daily living
BMI: body mass index
BODE: body mass index, airflow obstruction, dyspnea, exercise
COPD: chronic obstructive pulmonary disease
CRQ: Chronic Respiratory Questionnaire
eDSMP: Internet-based dyspnea self-management program
fDSMP: face-to-face dyspnea self-management program
FEV1: forced expiratory volume in 1 s
FVC: forced vital capacity
PDA: personal digital assistant
SF-36: Medical Outcomes Study Short-Form 36
SOB: shortness of breath
