# Supplementary material for: Randomized Controlled Trial of an Internet-Based Versus Face-to-Face Dyspnea Self-Management Program for Patients With Chronic Obstructive Pulmonary Disease: Pilot Study
Source: J Med Internet Res. 2008 Apr 16;10(2):e9. doi: 10.2196/jmir.990 (PMC2483918; doi:10.2196/jmir.990)
Supplement: Supplementary file 1 [file jmir_v10i2e9_app1.ppt]

## Slide 1
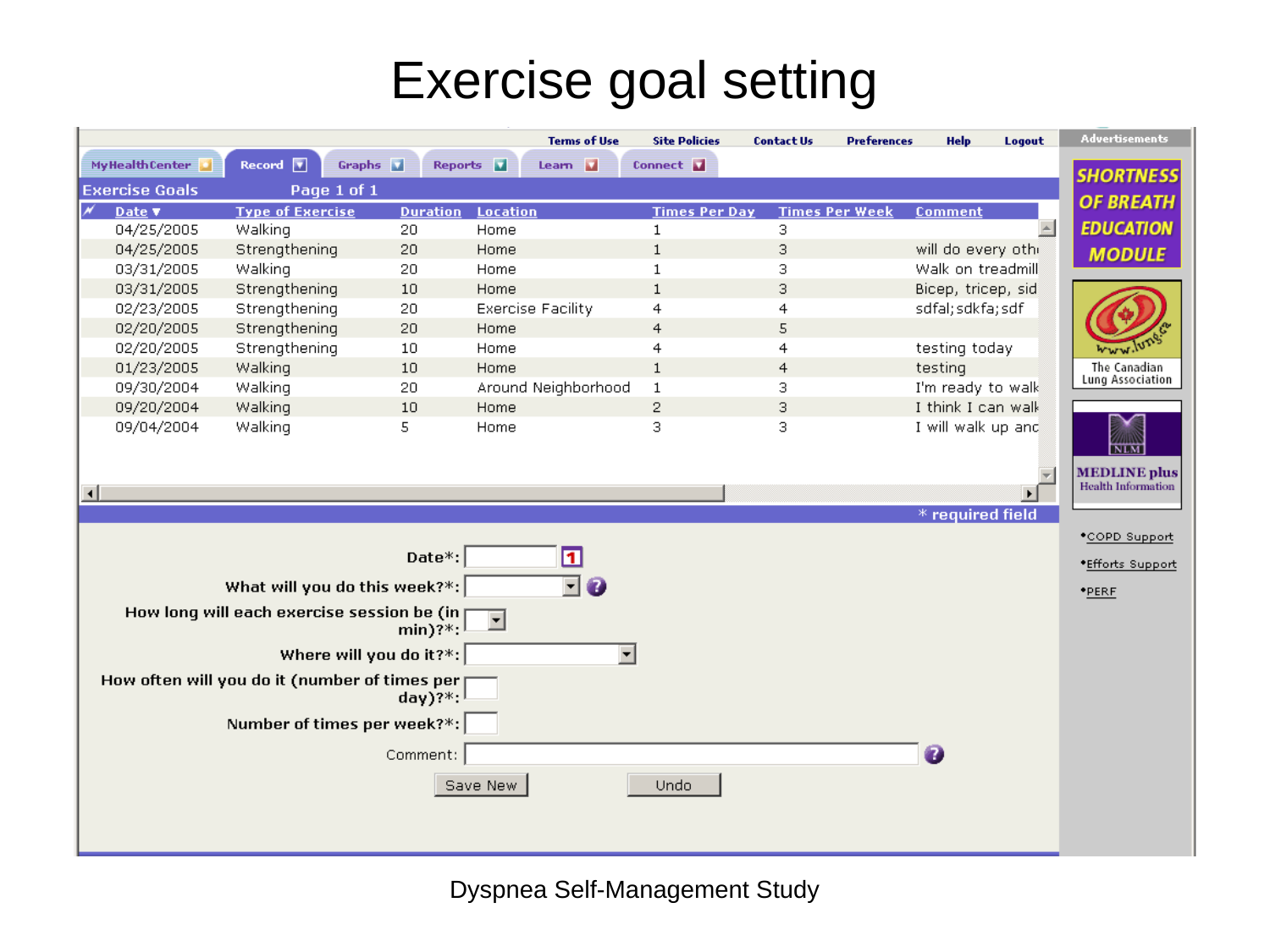

Exercise goal setting
Dyspnea Self-Management Study

## Slide 2
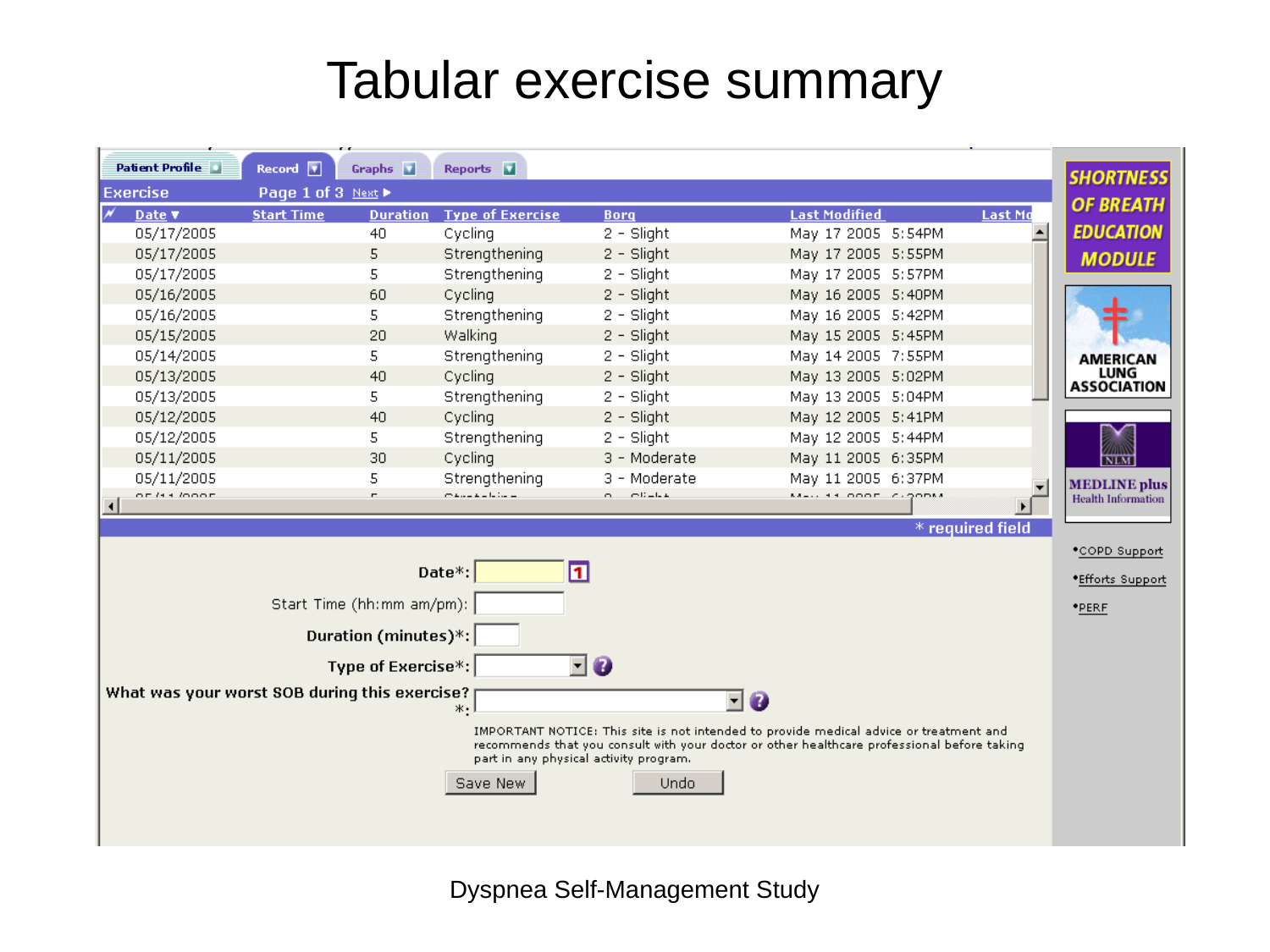

Tabular exercise summary
Dyspnea Self-Management Study

## Slide 3
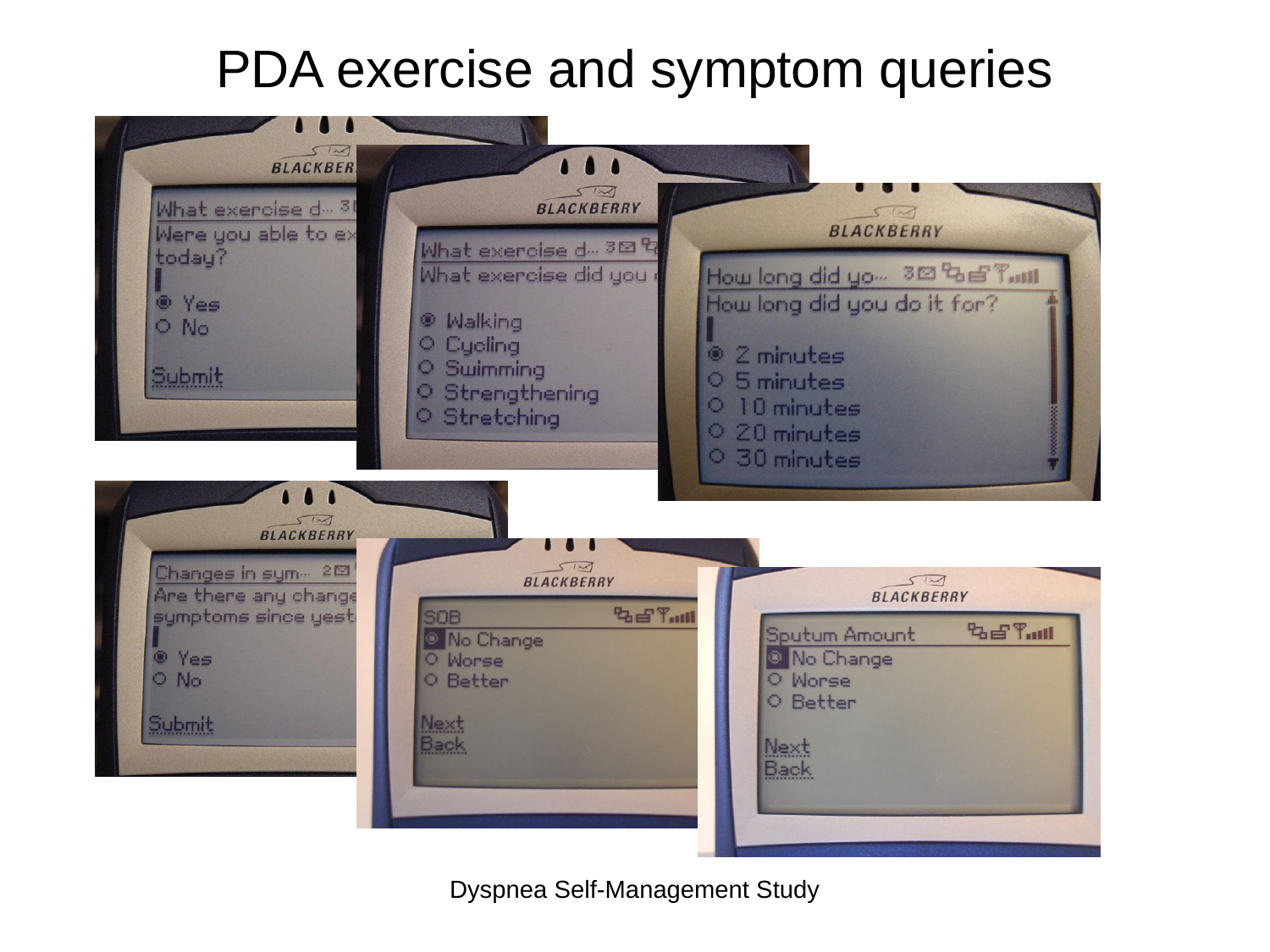

PDA exercise and symptom queries
Dyspnea Self-Management Study

## Slide 4
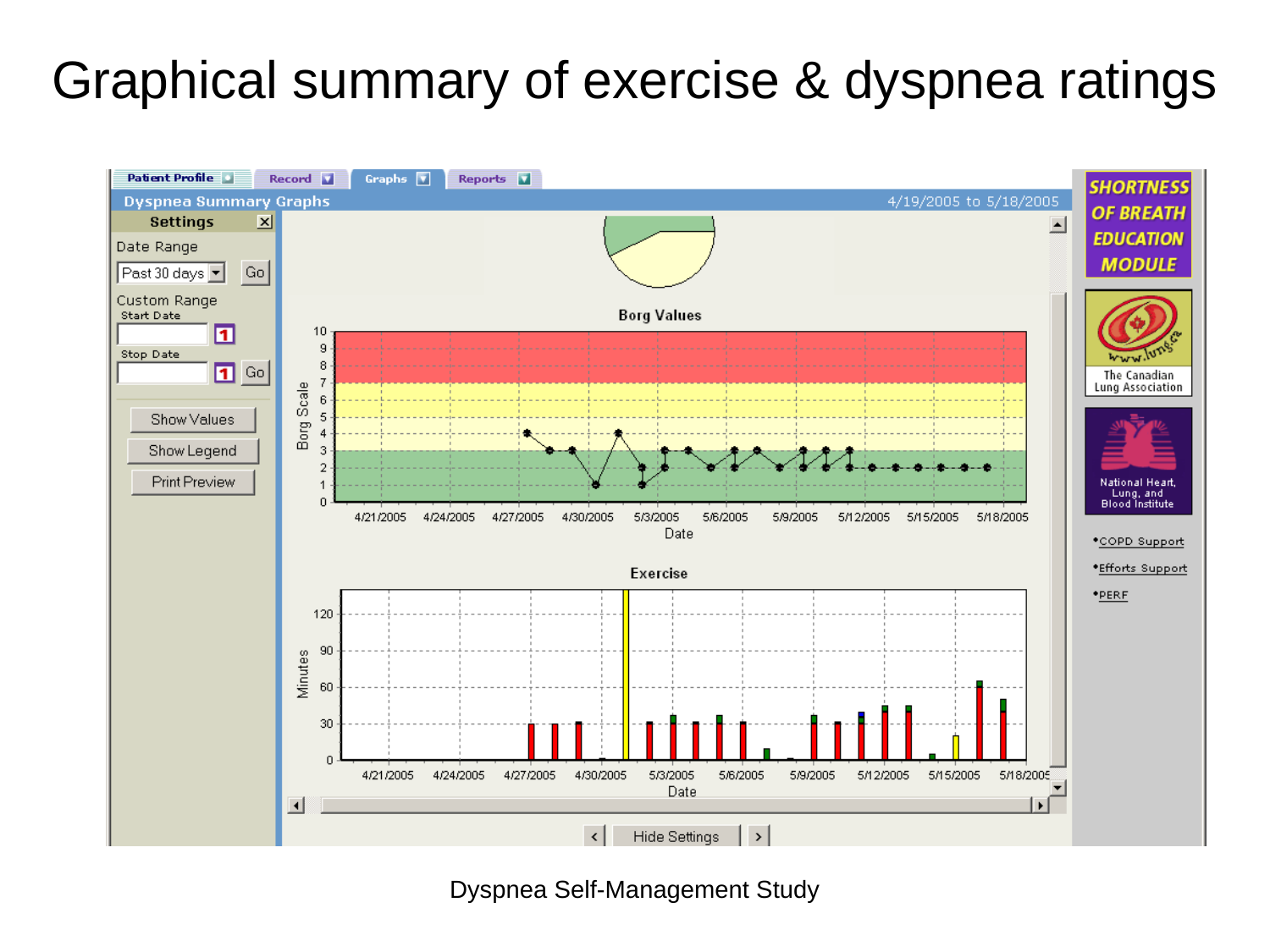

Graphical summary of exercise & dyspnea ratings
Dyspnea Self-Management Study

## Slide 5
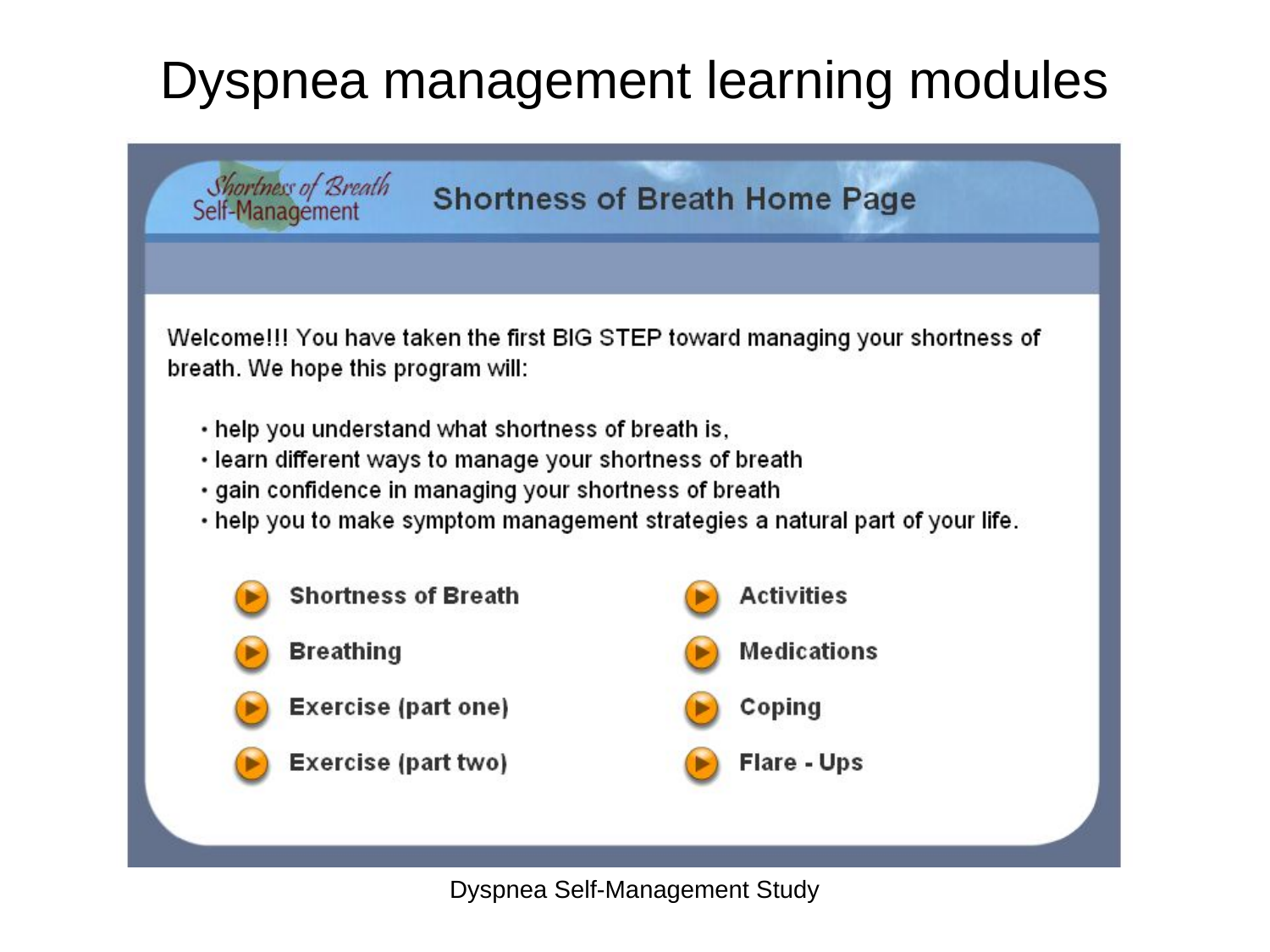

# Dyspnea management learning modules
Dyspnea Self-Management Study

## Slide 6
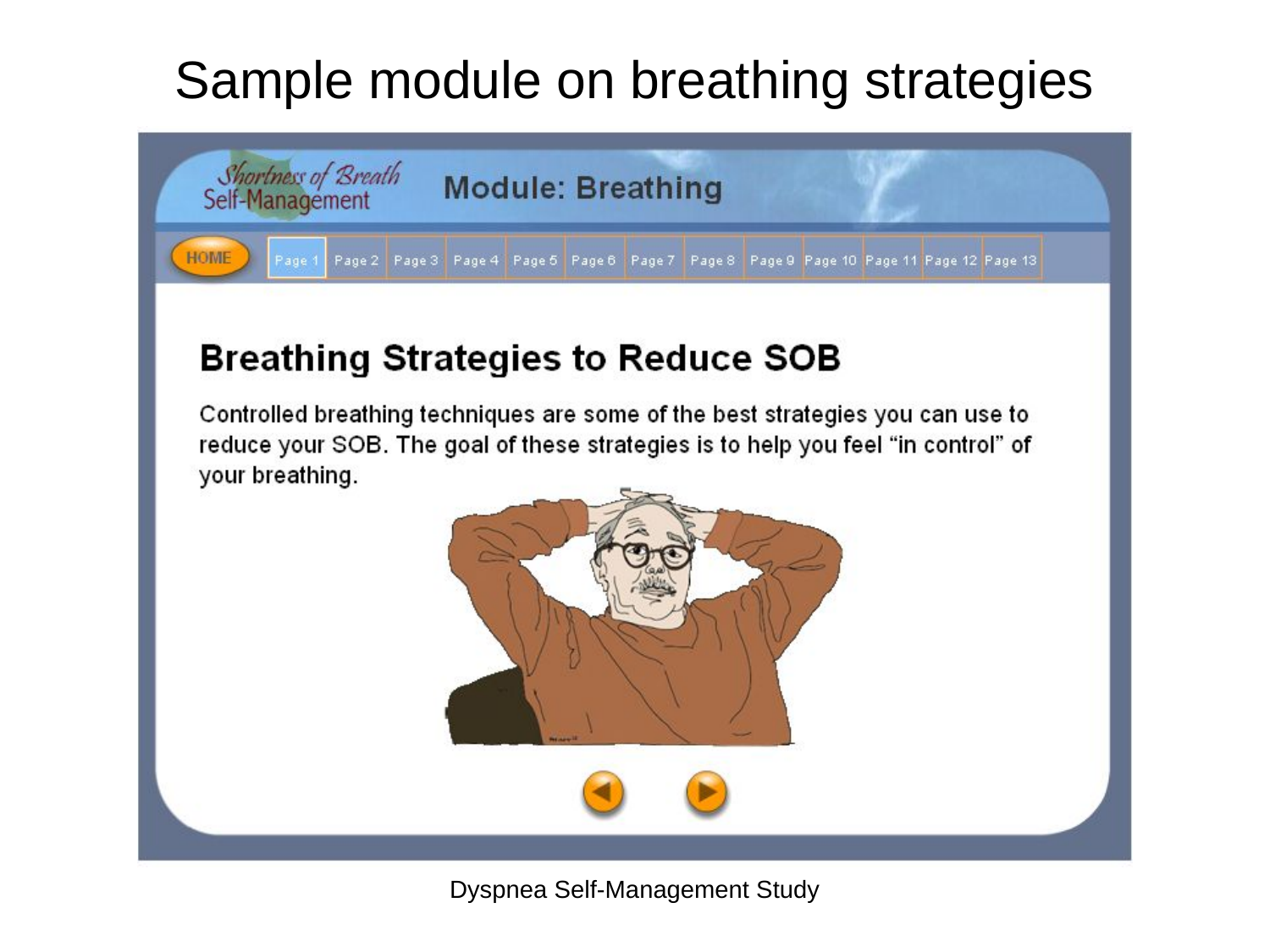

# Sample module on breathing strategies
Dyspnea Self-Management Study
